# Efficient Early Diagnosis of Sepsis Using Whole-Blood PCR–Reverse Blot Hybridization Assay Depending on Serum Procalcitonin Levels

**DOI:** 10.3389/fmed.2020.00390

**Published:** 2020-07-31

**Authors:** Seoyong Kim, Jungho Kim, Hyo Youl Kim, Young Uh, Hyeyoung Lee

**Affiliations:** ^1^Department of Biomedical Laboratory Science, College of Health Sciences, Yonsei University, Wonju, South Korea; ^2^Department of Clinical Laboratory Science, College of Health Sciences, Catholic University of Pusan, Busan, South Korea; ^3^Department of Internal Medicine, Yonsei University Wonju College of Medicine, Wonju, South Korea; ^4^Department of Laboratory Medicine, Yonsei University Wonju College of Medicine, Wonju, South Korea

**Keywords:** sepsis, PCR–reverse blot hybridization assay, blood culture, whole blood, procalcitonin, C-reactive protein

## Abstract

Sepsis is one of the medical emergencies, and its early detection, within the first hours of development, and proper management improve outcomes. Molecular diagnostic assays using whole blood collected from patients with suspected sepsis have been developed, but the decision making is difficult because of the possibility of false positives, due to contamination. Here, we evaluated the performance of the reverse blot hybridization assay (REBA) Sepsis-ID test for the detection of sepsis-causing microorganisms using whole-blood samples. In addition, the concentrations of C-reactive protein (CRP) and procalcitonin (PCT) were determined to evaluate whether these biomarkers can provide criteria for performing REBA Sepsis-ID in clinical settings. For this study, EDTA-anticoagulated whole blood was simultaneously collected for REBA Sepsis-ID and blood culture from 440 patients with suspected sepsis, from January to October 2015. In addition, CRP and PCT concentrations were measured in 227 patients. The overall positive rates of REBA Sepsis-ID and blood culture were 16.6% (73/440) and 13.9% (61/440), respectively. The pathogen-positive rates of REBA Sepsis-ID and blood culture were 9.8% (43/440) and 9.5% (42/440), respectively. The areas under the receiver operating characteristic (AUROC) curves of PCT and CRP for predicting pathogen-positive results of REBA Sepsis-ID were 0.72 and 0.69, respectively. The PCT concentrations in the group of patients aged ≥50 years were significantly higher than those in the group aged <50 years. After adjusting for age, the PCT AUROC value was 0.77 for predicting pathogen-positive results of REBA Sepsis-ID. The optimal cutoff values of PCT concentrations for subsequent application of REBA Sepsis-ID were 0.12 ng/mL in all patients and 0.22 ng/mL in patients aged ≥50 years. Our observations showed that REBA Sepsis-ID using whole blood was advantageous for the early detection of sepsis-causing microorganisms, and the PCT concentration could be used to determine the necessity of using REBA Sepsis-ID in clinical settings. The application of REBA Sepsis-ID using whole blood, based on the PCT concentration, may contribute to a highly efficient detection of sepsis-causing microorganisms.

## Introduction

Numerous studies have reported that the timely antimicrobial therapy can improve the outcomes in patients with sepsis. An observational study by Kumar et al. (1) showed an association between the delay in the initiation of antimicrobial therapy and mortality, with a 7.6% elevation in the risk of mortality for every hour of delay. Accordingly, the Sepsis Surviving Campaign guidelines recommend the administration of effective intravenous antimicrobials within the first hour of the recognition of septic shock or severe sepsis without septic shock as the goal of therapy, with daily reassessment of the antimicrobial regimen for potential de-escalation (2). Although effective, this treatment can destroy the human body's normal microbiota by indiscriminately attacking both virulent and avirulent or beneficial microorganisms (3). In addition, repeated administration of broad-spectrum antimicrobials can lead to the development of antimicrobial resistance and antibiotic-associated *Clostridioides difficile* infection (4). Therefore, rapid identification (ID) and an antimicrobial susceptibility test (AST) of sepsis-causing microorganisms play critical roles in the optimal treatment of patients, as broad-spectrum antimicrobial agents should be changed to narrow-spectrum agents, which are only effective against specific microorganisms. However, the turnaround time (TAT) of conventional microbiological procedures is 1–3 days from the time of sample collection to obtaining Gram staining, microbial ID, and AST results (5). Therefore, optimization of the laboratory workflow, technological innovations such as rapid AST platforms, and further laboratory automation are required for reducing the microbiological TAT (5).

Molecular diagnostics platforms have been developed for direct testing of patient's whole blood, without the need of culture. These include SeptiFast (Roche Diagnostics, Mannheim, Germany), using multiplex real-time polymerase chain reaction (PCR) coupled with probe hybridization; T2Candida and T2Bacteria (T2Biosystems, Lexington, MA, USA), using PCR–magnetic resonance; Iridica Plex ID (Abbott Molecular, Des Plaines, IL, USA), using PCR–electrospray ionization mass spectrometry; SepsiTest (Molzym, Bremen, Germany), using PCR–sequencing; MagicPlex Sepsis (Seegene, Seoul, Republic of Korea), using multiplex PCR; and REBA Sepsis-ID (Optipharm, Osong, Republic of Korea), using PCR–reverse blot hybridization assay (6, 7). These assays allow ID of pathogens within 3–12 h and require a blood volume of 5 mL or less for testing (6–9). Although rapid, these assays have some limitations. Several studies have highlighted the requirement of strategies for differentiating between true infections and contaminations and between patients with sepsis and individuals with transient bloodstream infections (8, 10). In addition, true-positive results of molecular assays are only confirmed in a low proportion of patients for whom microbiological tests are performed (11). Furthermore, these molecular assays are expensive and require special equipment and technical expertise (12). Issues such as a low detection rate of pathogens in the blood of patients with sepsis and other limitations hinder the use of these molecular assays for all patients for whom blood culture (BC) is required (13).

Recently, the use of sepsis-associated biomarkers has been recommended to compensate for the limitations of molecular diagnostic assays. Mihajlovic et al. (14) reported that presepsin and procalcitonin (PCT) concentrations can predict SeptiFast results and assist in selecting patients who require the SeptiFast test. In this study, we aimed to evaluate the performance of REBA Sepsis-ID using whole-blood samples collected from patients with suspected sepsis. Furthermore, we investigated whether PCT and C-reactive protein (CRP) concentrations could assist in decision making regarding the use of REBA Sepsis-ID in clinical settings.

## Materials and Methods

### Study Population

This prospective study was conducted at a tertiary care university hospital in Wonju, Republic of Korea. All adult patients who were admitted to the emergency room between January and October 2015 were screened. Patients were enrolled consecutively and prospectively if the criteria of systemic inflammatory response syndrome (SIRS), with suspected bacterial or fungal infection were met. As a result, a total of 440 adult patients were enrolled in our study. To evaluate the performance of REBA Sepsis-ID, we carried out BC and REBA Sepsis-ID simultaneously, using whole blood collected from these 440 patients. SIRS was diagnosed when two or more of the following symptoms were present: (1) an abnormal body temperature (<36°C or >38°C); (2) tachycardia (>90 beats/min); (3) tachypnea (>20 breaths/min); and (4) abnormal white blood cells counts (<4 × 10^9^/L or >12 × 10^9^/L). This study was approved by the Institutional Ethics Committee of the Yonsei University Wonju College of Medicine (approval no. CR312055-022). Written informed consent was obtained from the participants aged 16 and over for this study. The study population was classified into three groups, namely, pathogen-positive, contaminant-positive, and pathogen/contaminant-negative patients, respectively.

### BC and Additional Microbiological Tests

Two or three pairs of BC bottles, for aerobes or anaerobes, were incubated in a BacT/Alert 3D (bioMérieux, Durham, NC, USA) or BACTEC FX (Becton Dickinson Diagnostic Systems, Spark, MD, USA) BC system for 5 days after inoculation with 5–10 mL (per bottle) of the blood drawn from the patients. Conventional ID and ASTs were performed using the MicroScan system (Beckman Coulter, Brea, CA, USA) or Vitek® 2 system (bioMérieux).

### DNA Preparation

DNA was extracted from 1 mL of whole blood, which was incubated with 3 mL of erythrocyte lysis buffer (ELB) (Sigma–Aldrich, St. Louis, MO, USA) at room temperature for 10 min to disrupt erythrocytes. The supernatant was removed after centrifugation at 13,000 × g at 20°C for 5 min. The pellet was washed with 1 mL of ELB to completely remove erythrocytes and then centrifuged under the same conditions. The pellet was resuspended in 100 μL of a 5% Chelex 100 resin (Bio-Rad, Hercules, CA, USA) solution (in 1 × Tris-EDTA buffer, pH 8.0), and the suspension was then frozen and thawed twice, followed by boiling for 15 min in a heat block (FINEPCR, Daejeon, Republic of Korea). After centrifugation at 13,000 × g at 4°C for 10 min, the supernatant was used as the DNA template for REBA Sepsis-ID.

### REBA Sepsis-ID

REBA Sepsis-ID (Optipharm) was performed according to the manufacturer's instructions. PCR was performed in a volume of 20 μL, containing 10 μL of 2 × PCR master mix (GeNet Bio, Daejeon, Republic of Korea), 5 μL of 1 × biotinylated primer mix, and 5 μL of sample DNA. For DNA amplification, the first 15 cycles involved initial denaturation at 95°C for 30 s, followed by annealing and extension at 60°C for 1.5 min. These 15 cycles were followed by 35 cycles of denaturation at 95°C for 30 s, followed by annealing and extension at 54°C for 1 min. After the final cycle, the samples were maintained at 72°C for 10 min to complete the synthesis of all strands. For hybridization of the biotinylated amplicons, membrane strips containing a set of immobilized oligonucleotides were used. The oligonucleotides for REBA Sepsis-ID included 16 bacterial probes (gram-negative bacteria: *Escherichia coli, Shigella* spp., *Salmonella* spp./*Enterobacter* spp., *Klebsiella pneumoniae, Pseudomonas aeruginosa, Serratia* spp./*Morganella* spp., *Acinetobacter baumannii*, and *Haemophilus influenzae*; gram-positive bacteria: *Bacillus* spp., *Enterococcus* spp., *Streptococcus* spp., *Streptococcus pneumoniae, Staphylococcus* spp., and *Staphylococcus aureus*), five fungal probes (*Candida albicans, C. tropicalis, C. glabrata, C. parapsilosis*, and *C. krusei*), and three antibiotic resistance-associated probes (*mecA, vanA*, and *vanB*), as described by Wang et al. (7). The biotinylated amplicons were denatured at 25°C for 5 min in a denaturation solution and incubated with 1 mL of a hybridization solution on the REBA membrane strip in a blotting tray. The denatured, single-stranded biotinylated amplicons were hybridized with the probes on the strip at 55°C for 30 min. The strips were then washed twice in 1 mL of a washing solution at 55°C for 10 min with gentle shaking and incubated at 25°C for 30 min with a streptavidin–alkaline phosphatase conjugate (Roche Diagnostics) diluted to 1:2,000 in a conjugate diluent solution (CDS). Next, the strips were washed twice with 1 mL of CDS at room temperature for 1 min. Colorimetric hybridization signals were visualized after the addition of a staining solution with nitroblue tetrazolium-5-bromo-4-chloro-3-indolylphosphate (Roche Diagnostics) diluted to 1:50; the strips were incubated with the solution until the color was developed. Finally, the band patterns were read and interpreted.

### Evaluation of Clinical Relevance for Microorganisms

To determine whether the results of REBA Sepsis-ID and BC were true positive or not, the clinical relevance of the microorganisms identified was evaluated by two infectious disease specialists. Whether the microorganisms detected using REBA Sepsis-ID and BC represented true infections or not was evaluated retrospectively by considering the patient's clinical history, physical examination, body temperature, leukocyte count, underlying diseases, number of positive blood cultures out of the total number performed, and results of cultures of specimens from other sites, as defined previously (15–18). In the case of BC, the isolates were considered pathogens if at least two samples yielded the same microorganism. The isolates were considered contaminants if the skin microbiota such as coagulase-negative staphylococci, α-hemolytic streptococci, *Corynebacterium* spp., *Bacillus* spp., or *Propionibacterium acnes* was detected in only one sample, without other evidence of infection. The microorganisms detected using REBA Sepsis-ID were considered pathogens according to the criteria used by Leli et al. (19), as follows: (1) microorganisms identified using REBA Sepsis-ID and BC coincided as the cause of sepsis in the medical records; (2) microorganisms detected using only REBA Sepsis-ID coincided with the results of culture from the suspected infectious foci; (3) microorganisms detected using only REBA Sepsis-ID belonged to a species generally accepted as a common etiological agent of the patient's type of infection; (4) microorganisms detected by only one test were considered pathogens, if reported as the cause of the episode of sepsis in the final diagnosis, based on clinical, instrumental, and laboratory data. Coagulase-negative staphylococci and other Gram-positive bacteria identified using REBA Sepsis-ID were considered contaminants when isolated from only one set of BC (17), and in the absence of clinical and/or microbiological data suggesting their pathogenic role.

### Measurement of CRP and PCT Concentrations

Serum CRP concentrations were quantified using a cobas c 702 automated clinical chemistry analyzer (Roche, Basel, Switzerland). The assay was based on immunoturbidimetry, with the detection range of 0.03–350 mg/dL. Serum PCT concentrations were measured using VIDAS® B.R.A.H.M.S PCT™ (bioMérieux), with the detection range of 0.05–200 ng/mL. The patients with CRP or PCR values below the detection limit were excluded. In addition, the patients who did not receive the PCT testing were excluded. Of the 440 participants included in this study, 227 patients had data on the CRP and PCT concentrations at the BC sampling time.

### Statistical Analysis

Statistical analysis was performed using the GraphPad Prism 8 software version 8.0.2 (GraphPad Software, La Jolla, CA, USA) and the SPSS Statistics software version 25.0 (IBM, Armonk, NY, USA). One-way analysis of variance was performed to compare the PCT and CRP concentrations in the three groups of patients, who showed pathogen-positive, contaminant-positive, and negative results, respectively. Tukey's *post-hoc* test was performed to determine which pairwise comparisons are significant. The accuracy of PCT and CRP concentrations for predicting patients with pathogen infection was analyzed by calculating the area under the receiver operating characteristic (AUROC) curve. We also determined the sensitivity, specificity, positive predictive value (PPV), and negative predictive value (NPV) for each cutoff value to divide REBA Sepsis-ID results before and after adjustment for age.

## Results

### Comparison of Microbial ID Between BC and REBA Sepsis-ID Depending on Underlying Disease

In the 440 patients, positive rates of REBA Sepsis-ID and BC were 16.6% (73/440) and 13.9% (61/440), respectively. Of 73 patients with positive REBA Sepsis-ID results, 27 patients had underlying disease in respiratory system, 11 patients had renal system disease, 7 patients had gastrointestinal (GI) disease, 8 patients had sepsis/severe sepsis/septic shock, 5 patients had central nervous system (CNS) disease, 5 patients had complex disease, 4 patients had hepatobiliary disease, 2 patients had cancer, 2 patients had skeletal muscle (SM) disease, and 2 patients had other disease. Of 61 patients with positive BC results, 24 patients had respiratory disease, 9 patients had sepsis/severe sepsis/septic shock, 8 patients had renal system disease, 6 patients had complex disease, 3 patients had other disease, 2 patients had GI disease, 2 patients had cancer, 2 patients had SM disease, 2 patients had hepatobiliary disease, 1 patient had CNS disease, and 1 patient had skin disease (Table 1).

**Table 1:** Identification of microorganisms detected using blood culture and REBA Sepsis-ID.

**Underlying disease (*n*)**	**Number of cases**	**Microbial species**	
		**Blood culture**	**REBA Sepsis-ID**
Respiratory disease (172)	2	*Candida albicans*	*Candida albicans*
	2	*Staphylococcus aureus*	*Staphylococcus aureus*
	1	*Acinetobacter baumannii*	*Acinetobacter baumannii*
	1	*Escherichia coli*	Gram-negative bacteria
	1	*Staphylococcus hominis*	*Staphylococcus* spp.
	1	*Stenotrophomonas maltophilia*	Gram-negative bacteria
	1	*Acinetobacter baumannii* *Klebsiella pneumoniae*	*Streptococcus pneumoniae*
	1	*Corynebacterium striatum* *Enterococcus faecium*	Gram-positive bacteria
	1	*Staphylococcus epidermidis*	*Streptococcus* spp. *Staphylococcus* spp.
	4	*Stenotrophomonas maltophilia*	Not detected
	1	*Acinetobacter baumannii*	Not detected
	1	*Bacillus* spp.	Not detected
	1	*Candida tropicalis*	Not detected
	1	*Chryseobacterium meningosepticum*	Not detected
	1	*Enterococcus faecium*	Not detected
	1	*Propionibacterium acnes*	Not detected
	1	*Staphylococcus aureus*	Not detected
	1	*Staphylococcus epidermidis*	Not detected
	1	*Staphylococcus hominis*	Not detected
	3	Not detected	*Staphylococcus* spp.
	2	Not detected	Gram-positive bacteria
	2	Not detected	Gram-negative bacteria
	2	Not detected	*Streptococcus pneumoniae*
	1	Not detected	*Acinetobacter baumannii*
	1	Not detected	*Candida tropicalis*
	1	Not detected	*Escherichia coli*
	1	Not detected	*Staphylococcus aureus*
	1	Not detected	*Staphylococcus* spp.
	1	Not detected	*Candida parapsilosis* *Pseudomonas aeruginosa*
	1	Not detected	*Klebsiella pneumoniae* *Streptococcus pneumoniae*
	132	Not detected	Not detected
Gastrointestinal disease (62)	1	*Acinetobacter baumannii*	*Acinetobacter baumannii*
	1	*Candida albicans*	*Candida albicans*
	2	Not detected	Gram-positive bacteria
	1	Not detected	*Staphylococcus* spp.
	1	Not detected	*Staphylococcus aureus*
	1	Not detected	*Streptococcus* spp.
	55	Not detected	Not detected
Central nervous system disease (41)	1	*Staphylococcus aureus*	*Streptococcus* spp.
	3	Not detected	*Staphylococcus* spp.
	1	Not detected	*Streptococcus* spp. *Candida albicans*
	36	Not detected	Not detected
Renal system disease (38)	2	*Escherichia coli*	Gram-negative bacteria
	1	*Escherichia coli*	*Escherichia coli*
	1	*Staphylococcus epidermidis*	*Staphylococcus* spp.
	1	*Escherichia coli* *Enterococcus faecalis*	*Escherichia coli* *Enterococcus* spp.
	1	*Propionibacterium acnes*	Not detected
	1	*Staphylococcus capitis*	Not detected
	1	*Staphylococcus hominis*	Not detected
	1	Not detected	Gram-positive bacteria
	1	Not detected	Gram-negative bacteria
	1	Not detected	*Staphylococcus* spp.
	1	Not detected	*Streptococcus* spp.
	1	Not detected	*Escherichia coli* *Staphylococcus* spp.
	1	Not detected	*Klebsiella pneumoniae* *Staphylococcus* spp.
	24	Not detected	Not detected
Cancer (18)	1	*Staphylococcus hominis*	*Staphylococcus* spp.
	1	*Escherichia coli*	Not detected
	1	Not detected	*Streptococcus* spp.
	15	Not detected	Not detected
Skeletal muscle disease (18)	1	*Candida albicans*	Not detected
	1	*Staphylococcus lugdunensis*	Not detected
	2	Not detected	*Staphylococcus* spp.
	14	Not detected	Not detected
Sepsis/severe sepsis/septic shock (16)	1	*Acinetobacter baumannii*	*Acinetobacter baumannii* *Staphylococcus* spp.
	1	*Escherichia coli*	*Escherichia coli*
	1	*Klebsiella pneumoniae*	*Klebsiella pneumoniae*
	1	*Staphylococcus epidermidis*	Gram-positive bacteria
	1	*Acinetobacter baumanniiEnterobacter aerogenesEnterococcus faecium*	*Acinetobacter baumannii*
	1	*Acinetobacter baumannii*	Not detected
	2	*Staphylococcus aureus*	Not detected
	1	*Enterococcus faecium* *Acinetobacter baumannii*	Not detected
	1	Not detected	*Acinetobacter baumannii*
	1	Not detected	Gram-positive bacteria
	1	Not detected	*Staphylococcus aureus*
	4	Not detected	Not detected
Hepatobiliary disease (14)	1	*Escherichia coli*	Gram-negative bacteria
	1	*Stenotrophomonas maltophilia*	Not detected
	1	Not detected	*Escherichia coli*
	1	Not detected	Gram-positive bacteria
	1	Not detected	*Klebsiella pneumoniae*
	9	Not detected	Not detected
Skin/soft tissue disease (6)	1	*Staphylococcus capitis*	Not detected
	5	Not detected	Not detected
Viral infection (3)	3	Not detected	Not detected
Others (29)	2	*Staphylococcus hominis*	Not detected
	1	*Escherichia coli*	Not detected
	1	*Staphylococcus epidermidis*	Not detected
	1	Not detected	*Candida albicans*
	1	Not detected	*Streptococcus* spp.
	23	Not detected	Not detected
Complex disease (22)	1	*Staphylococcus capitis*	*Staphylococcus* spp.
	1	*Staphylococcus aureus*	*Staphylococcus aureus*
	1	*Escherichia coli*	*Escherichia coli*
	1	*Enterococcus faecalis*	Not detected
	1	*Stenotrophomonas maltophilia*	*Staphylococcus aureus*
	1	*Streptococcus anginosus*	Not detected
	1	Not detected	*Staphylococcus* spp.
	15	Not detected	Not detected

Among the underlying diseases, respiratory disease (172/440) was the most frequent, followed by gastrointestinal disease (62/440), central nervous system disease (41/440), renal system disease (38/440), cancer (18/440), skeletal muscle disease (18/440), sepsis/severe sepsis/septic shock disease (16/440), hepatobiliary disease (14/440), skin/soft tissue disease (6/440), and viral infection (3/440) (Table 1).

### Detection Rates of Pathogen/Contaminant by BC and REBA Sepsis-ID

Of the 61 cases with positive BC results, 42 cases were considered positive for pathogens, and the remaining 19 cases were considered positive for contaminants. Of the 73 cases with positive REBA Sepsis-ID results, 43 cases were considered positive for pathogens, and the remaining 30 cases were considered positive for contaminant. In the present study, there were a total of 74 discordant cases between BC and REBA Sepsis-ID. Of these, 31 cases produced positive BC and negative REBA Sepsis-ID results, while the remaining 43 cases yielded negative BC and positive REBA Sepsis-ID results (Table 2).

**Table 2:** Concurrence between the results of blood culture and REBA Sepsis-ID regarding clinical relevance.

**Blood culture**	**REBA Sepsis-ID**			
	**Pathogen**	**Contaminant**	**Negative**	**Total**
Pathogen	24	0	18	42
Contaminant	0	6	13	19
Negative	19	24	336	379
Total	43	30	367	440

### CRP and PCT Concentration in Patients According to the Clinical Relevance of the Microorganisms

Among the three groups based on the REBA Sepsis-ID results, the CRP concentrations were significantly higher in the pathogen-positive group than in the contaminant-positive (*p* < 0.05) and negative groups (*p* < 0.05), and the AUROC value of CRP was 0.69 in predicting cases with pathogens. Likewise, the PCT concentrations were significantly higher in the pathogen-positive group than in the other two groups, and the AUROC value of PCT was 0.72 in predicting cases with pathogens. Among the three groups based on the BC results, there were no significant differences in the CRP concentrations, and the AUROC value of CRP was 0.64 in predicting cases with pathogens. The PCT concentrations were significantly higher in the pathogen-positive group than in the other two groups, and the AUROC value of PCT was 0.72 in predicting cases with pathogens (Table 3).

**Table 3:** Concentrations of C-reactive protein (CRP) and procalcitonin (PCT) in participants according to clinical relevance of microorganisms detected using REBA Sepsis-ID and blood culture.

**Marker**	**REBA Sepsis-ID**					**Blood culture**				
	**Pathogen (mean ± SD, *n* = 23)**	**Contaminant (mean ± SD, *n* = 16)**	**Negative (mean ± SD, *n* = 188)**	***p*-value**	**AUROC value**	**Pathogen (mean ± SD, *n* = 28)**	**Contaminant (mean ± SD, *n* = 8)**	**Negative (mean ± SD, *n* = 191)**	***p*-value**	**AUROC value**
CRP (mg/dL)	16.0 ± 10.5	13.5 ± 11.4	10.5 ± 9.3	0.021	0.69	14.5 ± 10.1	11.5 ± 12.4	10.8 ± 9.5	0.177	0.64
PCT (ng/mL)	36.6 ± 63.0	7.7 ± 11.9	8.1 ± 22.7	<0.001	0.72	33.5 ± 59.5	3.0 ± 5.9	8.0 ± 21.8	<0.001	0.72

AUROC, area under the receiver operating characteristic curve; SD, standard deviation.

*p-values were calculated using one-way analysis of variance*.

### Age Adjustment and Cut-Off Values for PCT Concentration

In present study, there was significant difference in the PCT concentration according to patient's age (*p* = 0.03, Table 4). Furthermore, the PCT concentrations were significantly higher in the patients aged ≥50 years than in those aged <50 years, while there was no significant difference in the CRP concentrations between the two age groups. To determine whether the significant difference of PCT concentrations is just age-dependent or not, we further compared between two age group with negative REBA Sepsis-ID and BC results. Similar results were obtained when comparing patients from the two age group with negative REBA Sepsis-ID and BC results (Table 5).

**Table 4:** Concentrations of C-reactive protein (CRP) and procalcitonin (PCT) among age groups.

**Marker**	**Age**						
	**≤30** **(mean ± SD, *n* = 10)**	**40–49** **(mean ± SD, *n* = 22)**	**50–59** **(mean ± SD, *n* = 45)**	**60–69** **(mean ± SD, *n* = 32)**	**70–79** **(mean ± SD, *n* = 87)**	**≥80** **(mean ± SD, *n* = 31)**	***p*-value**
CRP (mg/dL)	8.9 ± 6.7	9.1 ± 7.8	12.5 ± 11.6	9.2 ± 9.4	12.0 ± 8.9	11.9 ± 11.0	0.49
PCT (ng/mL)	3.5 ± 8.1	1.3 ± 3.1	11.8 ± 41.9	15.2 ± 36.0	13.1 ± 29.4	8.4 ± 14.2	0.03

SD, standard deviation.

p-values were calculated using one-way analysis of variance.

**Table 5:** Concentrations of C-reactive protein (CRP) and procalcitonin (PCT) in participants after adjusting for age.

**Marker**	**All participants**			**Participants with negative results (both methods)**			**Participants with positive REBA Sepsis-ID or BC results**		
	**Age (years)**			**Age (years)**			**Age (years)**		
	** <50** **(mean ± SD,** ***n* = 32)**	**≥50** **(mean ± SD,** ***n* = 195)**	***p*-value**	** <50** **(mean ± SD,** ***n* = 21)**	**≥50** **(mean ± SD,** ***n* = 149)**	***p*-value**	** <50** **(mean ± SD,** ***n* = 11)**	**≥50** **(mean ± SD,** ***n* = 46)**	***p*-value**
CRP (mg/dL)	9.0 ± 7.4	11.6 ± 10.0	0.16	9.5 ± 8.1	10.5 ± 9.5	0.81	8.1 ± 6.1	15.3 ± 10.7	0.03
PCT (ng/mL)	2.0 ± 5.2	12.4 ± 32.0	0.02	1.2 ± 3.0	9.1 ± 24.1	0.03	3.2 ± 7.7	31.1 ± 56.5	0.02

SD, standard deviation.

p-values were calculated using Mann-Whitney test.

After adjusting for age, the AUROC value of PCT was 0.77 in predicting cases with true-positive REBA Sepsis-ID and BC results (Table 6). In this study, ~76% of participants were both BC and REBA Sepsis-ID negative. The PCT concentration can be a useful reference to rule out the negative results of REBA Sepsis-ID. Florkowski (20) mentioned that high sensitivity corresponds to high NPV and is ideal for a rule-out test. Therefore, the cut-off value with the highest sensitivity and NPV was selected. Before adjusting the results of REBA Sepsis-ID for age, at PCT concentrations >0.12 ng/mL, the sensitivity was 100.0% [95% confidence interval (CI): 85.2–100.0], the specificity was 19.2% (95% CI: 13.8–25.5), PPV was 13.1% (95% CI: 12.4–14.0), and NPV was 100.0%. After adjusting the results of REBA Sepsis-ID for age, at PCT concentrations >0.22 ng/mL, the sensitivity was 100.0% (95% CI: 85.2–100.0), the specificity was 32.5% (95% CI: 25.8–40.0), PPV was 10.9% (95% CI: 7.0–15.9), and NPV was 100.0% (Table 7).

**Table 6:** Concentrations of C-reactive protein (CRP) and procalcitonin (PCT) in participants ≥50 years old according to clinical relevance of microorganisms detected using REBA Sepsis-ID and blood culture.

**Marker**	**REBA Sepsis-ID**				**Blood culture**			
	**Pathogen (mean ± SD, *n* = 23)**	**Negative (mean ± SD, *n* = 188)**	***p*-value**	**AUROC value**	**Pathogen (mean ± SD, *n* = 23)**	**Negative (mean ± SD, *n* = 165)**	***p*-value**	**AUROC value**
CRP (mg/dL)	17.2 ± 10.7	10.8 ± 9.5	0.002	0.71	16.1 ± 10.2	11.0 ± 9.7	0.005	0.68
PCT (ng/mL)	42.0 ± 66.0	9.1 ± 24.2	<0.001	0.77	40.6 ± 63.6	8.9 ± 23.2	<0.001	0.77

AUROC, area under the receiver operating characteristic curve; SD, standard deviation.

*p-values were calculated using Mann-Whitney test*.

**Table 7:** Diagnostic parameters for procalcitonin concentrations before and after adjustment for age.

**Cutoff (ng/mL)**	**Parameter**			
	**Sensitivity, %** **(95% CI)**	**Specificity, %** **(95% CI)**	**PPV, %** **(95% CI)**	**NPV, %** **(95% CI)**
0.12	100.0 (85.2–100.0)	19.2 (13.8–25.5)	13.1 (12.4–14.0)	100.0
0.22^a^	100.0 (85.2–100.0)	32.5 (25.8–40.0)	10.9 (7.0–15.9)	100.0

a Age (≥50 years)-adjusted cutoff value.

CI, confidence interval; NPV, negative predictive value; PPV, positive predictive value.

## Discussion

Rapid ID of bloodstream pathogens is an important component of proper antimicrobial therapy and a positive outcome. In the absence of microbiological ID, empirical broad-spectrum antimicrobial treatment is used, which is associated with an increased risk of mortality, development of antimicrobial resistance, and high medical expenses (21–23).

Molecular methods for rapid ID of bloodstream pathogens have been developed to overcome the limitations of conventional BC, such as low sensitivity and the lack of rapid ID. The major advantage of using molecular methods is their rapid TAT (≤ 12 h) for reporting results, which is important for the management of patients with sepsis (13, 24–26).

In this study, we evaluated the utility of the molecular REBA Sepsis-ID method for the detection of sepsis-causing pathogens using whole blood collected from patients with sepsis (*n* = 440). Sepsis occurs when pathogens derived from the local infection enter bloodstream of patients (27). The lung infection is the most common cause followed by abdominal and urinary tract infections (28–31). In line with previous studies, our results showed among all patients with positive BC or REBA Sepsis-ID results, the most frequent underlying disease was respiratory disease. The overall positive rate of REBA Sepsis-ID using whole blood was 16.6%, which was higher than that of BC (13.9%). These data are consistent with those of other published studies, which compared BC and SeptiFast and showed that the positive rate of SeptiFast, the most widely evaluated molecular diagnostic assay, was higher than that of BC (32–35). However, Opota et al. (8) argued that, owing to their high sensitivity, molecular diagnostic assays are susceptible to contamination, and may yield false-positive results. Therefore, after the exclusion of possible non-causative microorganisms, the pathogen-positive rates of REBA Sepsis-ID and BC were estimated based on the evaluation of clinical relevance of microorganisms. The pathogen-positive rate of REBA Sepsis-ID (9.8%) was slightly higher than that of BC (9.5%). Similar results were reported by Westh et al. (32) and Burdino et al. (35), who confirmed higher pathogen-positive rates of SeptiFast than those of BC. As the detection of pathogens using BC is limited to living microorganisms, REBA Sepsis-ID may offer a potential advantage of detecting DNA of dead pathogens. In agreement with this, the literature provides evidence regarding higher pathogen-positive rates of SeptiFast than those of BC in patients treated with antimicrobials before sample collection (33, 36). In our study, no significant difference (*p* = 0.12) was observed in the positive rate between patients with or without antimicrobial therapy prior to BC (data not shown).

There are many possible reasons for the discordant results between REBA Sepsis-ID and BC. Basically, microorganisms detected vary depending on the type and characteristics of the probes used in REBA Sepsis-ID. An inadequate volume of blood collected for BC could contribute to the discordant (negative BC and positive REBA Sepsis-ID) results. However, there was no this case in our study. In addition, the discordant results can be occurred by the presence of viable but non-culturable (VBNC) bacteria found in central venous catheters (CVCs)-related infection (37). While the potential bias of REBA Sepsis-ID that detect DNA within/released from dead microorganisms might have affected the results (38). On the other hand, the positive BC and negative REBA Sepsis-ID results could be due to inhibiting factors present in the patient sample, or other, unidentified factors (39).

In our study, the rate of detection of contaminants using REBA Sepsis-ID (6.8%) was higher than that using BC (4.3%). The predominant contaminants detected using REBA Sepsis-ID were gram-positive bacteria, including *Staphylococcus* spp. and *Streptococcus* spp., which indicated a high risk of contamination by skin microorganisms such as staphylococci and streptococci. In a retrospective case-control study based on 254 false-positive results obtained using BC, Alahmadi et al. (40) demonstrated that incorrect diagnosis led to an increase in the length of hospital stay by 5.4 days (2.8–8.1), with an additional hospital cost of $1.6 million per year. In another study, contaminated BC increased patient charges by 47%, with an estimated cost of $8,720 per contamination (41). In the case of SeptiFast, a cutoff value is set to avoid false-positive results due to skin microbiota (32). Unfortunately, in the present study, there were no objective criteria for REBA Sepsis-ID, and therefore, clinical judgement must have been used to distinguish between false- and true-positive results.

At present, conventional diagnostic procedures take a few days. Specifically, blood culture can take 1–5 days to grow an organism to detectable levels, with additional time being required to identify and test for antimicrobial susceptibility (5). While, the REBA Sepsis-ID takes 3–4 h for the simultaneous detection of sepsis-causing pathogens and antimicrobial resistance-associated gene. Our study highlights a considerable reduction in the time required for the detection of sepsis-causing pathogens in whole blood using REBA Sepsis-ID. In particular, the use of REBA Sepsis-ID is useful for samples with negative BC results, which are routinely incubated for 5 days. Since a decrease in the time between the blood collection and the result reporting is associated with improved patient outcomes (5), a comparative study is needed on TAT of two techniques.

The costs associated with performing blood cultures were $36 per blood culture set and $104 for microorganism ID and AST (42). The estimated cost of REBA Sepsis-ID is $41 per sample, as referred to the cost of other line probe assay used in clinical microbiology laboratory such as the Genotype MTBDR assay (Hain Lifescience, Nehren, Germany) (43). The incorporation of REBA Sepsis-ID in diagnosis of patient with sepsis is likely to be highly cost-effective, although a reasonable cost-effectiveness analysis is needed.

Although REBA Sepsis-ID is a useful technique, it does have drawbacks. The procedure is relatively complicated due to post-PCR steps including hybridization, washing, and color development, and the requirement to have trained people for interpreting results. The post-PCR steps could be automated, including data interpretation (7). The PCR used in entire procedure is an extremely sensitive technique, which can lead to an increased risk of false positive results (44). In this regard, the REBA Sepsis-ID might be suitable for use at clinical laboratories where there is proven capacity to conduct molecular testing.

According to our results, PCT concentrations showed a good performance in predicting true-positive REBA Sepsis-ID results, with the AUROC value of 0.72. Our observations were similar to the results of another study, which evaluated the usefulness of PCT concentrations for the prediction of positive SeptiFast results, with the AUROC value of 0.75 (14). PCT concentrations also showed a good performance in predicting true-positive BC results, with the AUROC value of 0.72, similar to the results obtained in other studies, which confirmed a good discriminative power of PCT levels in predicting true-positive BC results (12, 45).

In addition, our results showed that the PCT concentrations in the participants aged ≥50 years were significantly higher than those in the participants aged <50 years, and the PCT AUROC value increased after adjusting for age. These findings may indicate age dependence of PCT concentrations in adult patients with suspected sepsis. Stocker et al. (46) reported that PCT concentrations of newborns increased during the first 2–3 days after birth and suggested that separate reference ranges should be applied to newborns with suspected sepsis during the first 48 h. However, there were no further reports on adult patients with sepsis. Our findings likely reflect the different prevalence of clinical conditions during a lifetime, some with lower inflammatory impact occurring at younger adults (47) and complex patient heterogeneity (i.e., surgery, medications) (48). In order to overcome these limitations in our study, higher-quality studies on the association between PCT concentrations and the age in adult patients with suspected sepsis are required for producing results that are closer to the true result (49) and selecting a well-defined cutoff for the PCT level to be used as a criterion for using REBA Sepsis-ID.

In this study, we also analyzed CRP concentrations as a possible predictive biomarker for true-positive REBA Sepsis-ID and BC results. The resulting CRP AUROC values were 0.69 for REBA Sepsis-ID and 0.64 for BC. Our observations were similar to the results of another study, which evaluated the use of CRP for predicting positive BC results, with the reported AUROC value of 0.65 (50). Although significant, CRP concentration showed a poor performance in predicting true-positive REBA sepsis-ID and BC results.

In addition to PCT and CRP, more than 100 different molecules have emerged as potentially useful biomarkers for sepsis (51, 52). Among these, CD64, a leukocyte surface antigen, has been described as a good candidate biomarker for sepsis. CD64 is constitutively expressed on neutrophils, albeit at low levels, in the absence of infection. The upregulation of CD64 on the cell surface of polymorphic mononuclear neutrophils is considered an early step in the innate immune response to bacterial infection (53, 54). Several studies have described the ability of neutrophil CD64 expression to differentiate between sepsis and non-sepsis cases, with a good diagnostic accuracy (AUROC values ranging from 0.92 to 0.95) (55–57). Therefore, further studies are necessary for investigating biomarkers that can reliably predict true-positive REBA Sepsis-ID results.

As the procedure for REBA Sepsis-ID is complicated and clinical experience is limited, with no clear explanations for false-positive and false-negative results, we suggest that the PCT concentration may be useful for determining the need to use the REBA Sepsis-ID test in clinical settings. Furthermore, efforts are necessary for improving the molecular techniques for detection of pathogens in whole blood, as several cases with positive BC results showed negative REBA Sepsis-ID results in this study. In addition, multicenter evaluation is required for comparing results among centers and increasing the generalizability of the study data. Only blood cultures were compared with the REBA Sepsis-ID. Thus, further studies are needed to examine the applicability of the REBA Sepsis-ID for detection of pathogens in various types of specimens such as urine and cerebrospinal fluid.

In conclusion, our results suggest that REBA Sepsis-ID may be implemented, using whole blood, as a useful add-on for patients with suspected sepsis, which requires rapid pathogen detection and identification. Furthermore, measurement of the PCT concentration prior to using REBA Sepsis-ID may be a good strategy for early and efficient detection of pathogens in the blood of patients with suspected sepsis.

## Data Availability Statement

All datasets generated for this study are included in the article/supplementary material.

## Ethics Statement

The studies involving human participants were reviewed and approved by Institutional Ethics Committee of the Yonsei University Wonju College of Medicine. Written informed consent to participate in this study was provided by the participants' legal guardian/next of kin.

## Author Contributions

SK designed and performed the experiments and wrote the manuscript. YU and HL contributed to all aspects of this research and revised the manuscript. JK performed the experiments and analyzed the data. HK analyzed the data and revised the manuscript. All authors contributed to the article and approved the submitted version.

## Conflict of Interest

The authors declare that the research was conducted in the absence of any commercial or financial relationships that could be construed as a potential conflict of interest.
